# Causes of prolonged fever among HIV infected patients- utility of an algorithmic approach - a single centre study from south India

**DOI:** 10.1186/1471-2334-14-S3-P15

**Published:** 2014-05-27

**Authors:** Sudheer Aree Parambil, Nitin Hosmelkar, Rajendra Prasad Shivaswamy

**Affiliations:** 1 Vivekananda Memorial Hospital, Saragur, Mysore, Karnataka, India; 2 ART centre, Hassan Institute of Medical Sciences, Hassan, Karnataka, India; 3 JSS medical college and Hospital, JSS University, Mysore, Karnataka, India

## Background

Availability of ART for the last one decade has changed the pattern of morbidity among HIV infected patients. Researchers from Christian Medical College Vellore had developed an algorithm for evaluation of prolonged fever in the pre HAART era in India. The purpose of this study was to assess utility of this algorithm in the current HAART era to identify causes of prolonged fever and correlate with CD4 count, WHO staging and ART status.

## Methods

Prospective longitudinal observational study conducted on convenient sample of 90 consecutive HIV infected patients presenting with prolonged fever at a secondary care hospital.

## Results

The algorithm developed by CMC, Vellore was useful in diagnosing causes of fever among 91.1% of the patients. TB meningitis was the most common cause of fever accounting for 23.3% followed by Bacterial pneumonia, Pulmonary TB and *Pneumocystis jiroveci* pneumonia accounting for 13.3%, 11.1% and 13.3% respectively. Immune reconstitution inflammatory syndrome and lymphoma were found to be the cause in 5.4% of patients

## Conclusion

Tuberculosis continues to be the most common cause of prolonged fever (58.8%) although the prevalence rates seems to be decreased compared to the pre HAART era studies from India (71%). Bacterial pneumonia was found to be increasing among all CD4 groups and regardless ART status pointing to the need for introduction of effective vaccination programs against *Pneumococcus*. CD4 count and WHO staging were found to be the statistically significant factors in determining the cause of fever and ART status was not statistically significant.

